# Complete Genome Sequences of Three Lactobacillus crispatus Strains Isolated from the Urine of Postmenopausal Women

**DOI:** 10.1128/MRA.01017-21

**Published:** 2021-12-02

**Authors:** Neha V. Hulyalkar, Belle M. Sharon, Brianna M. Shipman, Amanda P. Arute, Philippe E. Zimmern, Nicole J. De Nisco

**Affiliations:** a Department of Biological Sciences, University of Texas at Dallas, Richardson, Texas, USA; b Department of Urology, University of Texas Southwestern Medical Center, Dallas, Texas, USA; Loyola University Chicago

## Abstract

Lactobacillus crispatus frequently colonizes the vagina and bladder of healthy women. Although its association with vaginal health is relatively well understood, little is known about its role in urinary tract infection (UTI). Here, we report the complete genome sequences of three urinary L. crispatus strains isolated from women with different UTI histories.

## ANNOUNCEMENT

Lactobacilli are the most abundant members of the urinary microbiomes of heathy women; however, their role in maintaining bladder health is poorly understood (1–5). In the vagina, lactobacilli provide colonization resistance by maintaining an acidic pH and secreting antimicrobial compounds (5, 6). Urinary lactobacilli may act similarly to resist uropathogen colonization during urinary tract infection (UTI) (1, 7).

Although Lactobacillus crispatus is frequently found in the urinary microbiome, before this work only 11 complete genome sequences were available in the NCBI database. Furthermore, no complete genome sequences of L. crispatus isolated from urine were available. Complete urinary L. crispatus genome sequences will enable analyses of the genetic factors mediating adaptation to the bladder, especially mobile genetic elements. Here, we report the complete genome sequences of three L. crispatus strains isolated from the urine of three postmenopausal women who either had no clinical history of UTI or had a history of recurrent UTI (rUTI) but were not experiencing UTI at the time (Table 1), as part of an institutional review board-approved study (STU 032016-006 and MR 17-120).

**TABLE 1:** Accession numbers, assembly parameters, and isolate characteristics of three Lactobacillus crispatus isolates from postmenopausal women with different UTI histories

Strain	Host health state	BioSample accession no.	SRA accession no.^*a*^	No. of raw reads	No. of trimmed reads	Read *N*_50_ (bp)	Read depth (×)	GenBank accession no.	Type of contig (circular)	Total length (bp)	GC content (%)	No. of CDSs^*b*^
Lc116_C48	History of rUTI	SAMN21367957	SRR15987647 (O)	260,487	260,038	5,427	352	CP083393	Chromosome	2,350,925	37.2	2,304
			SRR16002670 (I)	5,740,374	5,671,944		330	CP083394	Plasmid	96,317	37.5	89
Lc1226_C128	History of rUTI	SAMN21367958	SRR15987646 (O)	201,546	201,213	4,789	240	CP083392	Chromosome	2,526,154	37.1	2,511
			SRR16002669 (I)	6,841,314	6,768,092		382					
Lc1700_C167	No UTI history	SAMN21367959	SRR15987645 (O)	225,675	225,180	4,455	220	CP083389	Chromosome	2,423,824	37.2	2,383
			SRR16002668 (I)	6,475,702	6,396,086		322	CP083390	Plasmid	200,485	34.5	178
								CP083391	Plasmid	194,655	34.5	181

a O, ONT; I, Illumina.

b Coding sequences.

Clean-catch midstream urine was obtained, plated onto De Man, Rogosa, and Sharpe (MRS) agar, and incubated for 3 days at 35°C in a microaerophilic atmosphere using the GasPak EZ Campy pouch system (BD). Species identification was performed by Sanger sequencing of the 16S rRNA gene and MegaBLAST (BLAST v2.10.0) (8). Well-isolated colonies were cultured in MRS broth for 3 days in a microaerophilic atmosphere at 35°C. Genomic DNA was extracted using the DNeasy blood and tissue kit (Qiagen) and purity analyzed using the 260/280 nm absorbance ratio and agarose gel electrophoresis (8).

The Nextera DNA Flex library prep kit was used for Illumina library preparation, and the NextSeq 500 was used to generate 2 × 150 bp paired-end reads. CLC Genomics Workbench v12.0.3 was used for Illumina read quality assessment and trimming, preserving reads with a Phred score below 20 and a minimum length of 15 bp.

Oxford Nanopore libraries were constructed using the ligation sequencing kit (SQK-LSK109) and barcode expansion kit 1-12 (EXP-NBD104) and sequenced on the MinION platform using R9 FLO-MIN106 flow cells. ONT MinKNOW software was used for live fast base calling, demultiplexing, and barcode trimming. NanoStats v1.2.0 and NanoFilt v2.6.0 were used for quality assessment and trimming, respectively, retaining reads of >200 bp with a Phred score of >7 (9).

Unicycler v0.4.8 (SPAdes v3.13.0, Racon v1.4.10, and Pilon v1.23) was used for hybrid assembly of the Illumina and ONT reads (10–14). Unicycler’s default mode generated closed assemblies for Lc1226_C128 and Lc1700_C167, whereas bold mode was required for Lc116_C48. The genomes were rotated to the start of the *dnaA* or *repA* gene, if found. QUAST v5.0.2 was used to assess the assembly quality (15). Bandage v0.8.1 and BUSCO v1 were used to determine the genome completeness using the bacteria ortholog set on the gVolante v1.2 server (16–18). The genomes were annotated using the NCBI Prokaryotic Genome Annotation Pipeline v4.11 (19, 20), while the GC content and coding sequence number were calculated using Geneious Prime v2020.0.5. All analysis parameters were default unless otherwise specified.

### Data availability.

The genome sequences are available in GenBank under BioProject accession number PRJNA761982. Table 1 displays the BioSample and SRA accession numbers for each isolate.
